# Evaluating tigecycline dosing for hospital-acquired pneumonia patients: insights from physiologically-based pharmacokinetic modeling of lung exposure

**DOI:** 10.1128/aac.00004-25

**Published:** 2025-05-20

**Authors:** Xiaonan Zhang, Feiyan Liu, Sanwang Li, Zeneng Cheng, Hanxi Yi, Feifan Xie

**Affiliations:** 1 Division of Biopharmaceutics and Pharmacokinetics, Xiangya School of Pharmaceutical Sciences, Central South University 506618, Changsha, China; 2 Department of Pharmacy, The Second Xiangya Hospital, Central South University 618805, Changsha, China; 3 Department of Pathology, School of Basic Medical Science, Central South University 618102, Changsha, China; Providence Portland Medical Center, Portland, Oregon, USA

**Keywords:** tigecycline, lung exposure, pneumonia, dosing regimen, PBPK modeling

## Abstract

Tigecycline is increasingly used off-label for hospital-acquired pneumonia (HAP), though its efficacy and optimal dosing remain uncertain. Lung exposure to tigecycline may be affected by pulmonary pH changes induced by bacterial infections. This study used a physiologically -based pharmacokinetic (PBPK) model to evaluate the impact of pH shifts on lung exposure and assess the efficacy of various dosing regimens. A lung PBPK model for tigecycline was developed and validated using plasma and lung concentration data from clinical pharmacokinetic studies. Simulations evaluated the impact of pH alterations from 6.6 (healthy) to 5.6 (infection) on lung exposure. Three clinical dosing regimens—standard (100 mg loading dose +50 mg q12h), median (150 mg loading dose +75 mg q12h), and high dose (200 mg loading dose +100 mg q12h)—were assessed by calculating the probability of target attainment (PTA) in lung compartments, including epithelial lining fluid (ELF) and alveolar cells (ACs), across a MIC range of 0.125–32 mg/L. The model reasonably captured tigecycline exposure in plasma and lung. Pulmonary pH alterations had minimal impact on tigecycline AUC in ELF but led to a significant 12.39-fold increase in AUC within ACs at pH 5.6. For pathogens with MIC ≤1 mg/L, all three dosing regimens achieved PTA ≥90% in ELF. However, for MIC >2 mg/L, only the high-dose regimen provided satisfactory PTA. The lung PBPK model provides valuable insights into tigecycline PK in HAP patients and underscores the need to optimize dosing for pneumonia with resistant pathogens.

## INTRODUCTION

Tigecycline, a third-generation tetracycline-class antibiotic, is initially indicated for the treatment of community-acquired pneumonia (CAP) caused by susceptible pathogens in adults (1, 2). However, it is increasingly used off-label for the management of hospital-acquired pneumonia (HAP), particularly in cases involving multidrug-resistant (MDR) and extensively drug-resistant (XDR) pathogens, where it is regarded as one of the most important last-resort antibiotics for severe infections (3–9). Despite its widespread use, the clinical effectiveness of tigecycline in HAP remains controversial, with concerns regarding its association with increased all-cause mortality, especially in patients with ventilator-associated pneumonia (VAP) (10). Conversely, other studies have reported no significant difference in mortality between tigecycline and alternative treatments for HAP, further contributing to the ongoing debate about its safety and efficacy (11, 12).

The standard dosing regimen for tigecycline in adult patients consists of an initial dose of 100 mg, followed by 50 mg every 12 hours, administered via intravenous infusion over 30 to 60 minutes. However, alternative dosing strategies, such as high-dose regimens (e.g., a 200 mg loading dose followed by 100 mg every 12 hours), have been explored in an attempt to improve clinical outcomes. While some studies have failed to demonstrate clear benefits over standard dosing (13, 14), others suggested that high-dose tigecycline may reduce mortality in HAP patients, fueling the debate surrounding its optimal dosing and effectiveness (12, 15).

The observed discrepancies in tigecycline’s effectiveness in HAP could be due, in part, to the high heterogeneity of HAP patient populations, compounded by the increasing prevalence of antibiotic resistance. Another important factor that may influence the efficacy of tigecycline in HAP is its pharmacokinetic (PK) profile, particularly in lung tissue where the concentrations are more directly related to the therapeutic success than in plasma (16). Given the complex and varied characteristics of HAP patients, who are often immunocompromised, elderly, or post-surgical, there is significant heterogeneity in both their underlying pathologies and drug responses.

In the case of tigecycline, its lipophilic properties, combined with moderate basicity, are associated with pH-driven lysosomal sequestration (17), which can lead to high drug concentrations in lysosome-rich tissues, such as the lung (18, 19). This intrapulmonary penetration of tigecycline contributed to its application for treating pneumonia. However, in pneumonia patients with bacterial infection, one of the hallmark physiological alterations is the more acidic lung microenvironment due to the local inflammatory response (20), compounded further by changes in lung penetration (21), increased mucus production, and leaky capillaries (22). This pH shift, in particular, could impact the efficiency of lysosomal sequestration to tigecycline (23), thereby influencing its lung exposure, which is an essential factor for achieving therapeutic efficacy in HAP.

Techniques such as bronchoalveolar lavage (BAL) and microdialysis are commonly used to measure antibiotic concentrations in lung tissue (24). However, these methods are not routinely employed in clinical practice, especially in critically ill patients. In this context, *in silico* modeling approaches, particularly physiologically-based pharmacokinetic (PBPK) models, offer a promising alternative. These models incorporate biological, physiological, and drug-specific parameters to simulate the distribution of drugs within the body, allowing for predictions of lung drug concentrations without the need for invasive measurements. Previous studies have successfully applied the mechanistic lung PBPK model to predict the lung exposure of several drugs and demonstrate that the accumulation in the lungs for some basic drugs is sensitive to changes in pulmonary pH (25–27).

Therefore, in this study, we propose to use a PBPK model to simulate tigecycline’s pharmacokinetics in the lungs of HAP patients, with the aim of evaluating the impact of pulmonary pH alterations on tigecycline lung exposure and assessing the potential efficacy of different dosing regimens based on tigecycline’s lung exposure. This modeling approach will provide valuable insights into the relationship between patient physiology and tigecycline lung exposure and facilitate the optimization of tigecycline dosing strategies for HAP patients.

## MATERIALS AND METHODS

### Clinical PK data for tigecycline

Clinical PK studies on tigecycline, including those in healthy volunteers and pneumonia patients, were identified in the literature up to June 30, 2024. A comprehensive search was conducted on PubMed using the following terms: ((pharmacokinetic [Title/Abstract]) OR (pharmacokinetics [Title/Abstract])) AND (tigecycline [Title/Abstract]). Studies were included if they met the following criteria: (i) clearly reporting dosing regimens and demographic data and (ii) providing tigecycline plasma, epithelial lining fluid (ELF), and/or alveolar cells (ACs) concentration data at either the population or individual level in healthy adults, or providing tigecycline plasma, ELF, and/or ACs concentrations in adult pneumonia patients without renal or hepatic impairment. Concentration–time profiles of tigecycline in plasma and/or lung were extracted from the selected publications and converted into a digital format using WebPlotDigitizer software (version 4.8, https://apps.automeris.io/wpd4/).

### Tigecycline PBPK model development and verification

#### Development of the basic tigecycline PBPK model

Parameters of the basic tigecycline PBPK model were primarily obtained from public databases and clinical data. Key physicochemical properties of tigecycline, such as log*P,* were obtained from the DrugBank (https://go.drugbank.com), while pKa and unbound fraction in plasma (*f*_*u*_) were taken from related literatures (17, 28). The distribution of tigecycline was modeled using a full PBPK distribution model, with the volume of distribution at steady state (*V*_ss_) predicted using the Rodgers and Rowland method (29). An optimized blood/plasma concentration ratio (B/P) of 1.3 was applied, yielding a *V*_ss_ prediction of 7.97 L/kg, which aligned with clinical observation (30). Total clearance (CL) of tigecycline was calculated to be 23.8 L/h based on a multiple-dose study in healthy adults (31). Tigecycline is primarily eliminated through bile excretion (59%), with additional elimination via renal excretion (22%) and hepatic metabolism (19%) (32). The intrinsic clearance for bile excretion and liver metabolism was estimated using retrograde modeling methods implemented in Simcyp (33), with values of 7.579 µL/min/million cells and 6.313 µL/min/mg protein, respectively. Renal clearance was calculated to be 5.236 L/h. The final input parameters for the basic tigecycline PBPK model are summarized in Table 1. The model was constructed using the Simcyp population-based simulator (version 21; Certara, Sheffield, UK).

**TABLE 1:** Final parameters for tigecycline PBPK model^*a*^

Parameter	Model input value	Sources/comments
Physicochemical data and blood binding		
Molecular weight (g/mol)	585.65	DrugBank
log *P*	0.8	DrugBank
Compound type	Ampholyte	
pKa1 (acid)	3.3	Reported (17)
pKa2 (base)	9.7	Reported (17)
Blood/plasma concentration ratio	1.3	Optimized
Unbound fraction in plasma	0.2	Reported (28)
Plasma binding component	Albumin	
Distribution		
Distribution model	Full PBPK model	
Volume of distribution at steady state (L/kg)	7.97	Predicted via Rodgers and Rowland method
Elimination		
Clearance model	Enzyme kinetics	
CL_int_ (Bile) (μL/min/million cells)	7.58	Calculated via retrograde model
CL_int_ (HLM) (μL/min/mg protein)	6.31	Calculated via retrograde model
CL_renal_ (L/h)	5.24	Assigning 22% of total Clearance (32)
Pulmonary parameters		
Henry’s constant (Pa m^3^/mol)	2.96E-24	Predicted via EPI Suite (v4.11)
Effective passive permeability (10^−4^ cm/s)	1.98E + 05	Estimated
Unbound fraction in pulmonary mass	0.016	Estimated
Unbound fraction in pulmonary fluid	1.0	Assumed
CL_int_ (apical efflux transporter) (μL/min/cm^2^)	2.5	Optimized

^*a*^ CL_int_: intrinsic clearance; HLM: human liver microsomes; CL_renal_: renal clearance.

#### Verification of the basic tigecycline PBPK model

The basic tigecycline PBPK model was preliminarily verified by comparing simulated concentration–time profiles in plasma and PK parameters with observed clinical data from single- and multiple-dose studies in healthy adults. For each clinical scenario, a virtual population of 100 subjects was generated, with 10 trials, each containing 10 subjects, to account for interindividual variability. The study design (e.g., dosing regimens) and population demographics (including age distribution and gender ratio) were matched to the corresponding clinical studies. Model performance was deemed satisfactory if the observed mean or individual plasma concentration data fell within the 90% prediction interval of the simulated concentration–time profiles. Furthermore, the predictive accuracy of mean PK parameters, such as peak concentration (*C*_max_) and area under the concentration–time curve (AUC), was assessed using a 0.5- to 2-fold range relative to observed values. This verification process and performance criterion were applied consistently across all PBPK models developed in this study.

#### Development of the multicompartment permeability-limited lung PBPK model for tigecycline

The lung PBPK model of tigecycline was extended based on the basic model, with the lung described in seven segments, including upper and lower airways (two segments) and the lobes of the lung (five segments). Each segment consisted of four compartments representing pulmonary capillary blood, tissue mass, fluid, and alveolar air. The fluid compartment was used to represent mucus and epithelial lining fluid, while the tissue mass compartment represented various cell types within the lung (26). Equilibrium was assumed between the fluid and alveolar air compartments, with the air:fluid partition coefficient (Henry’s constant) for tigecycline estimated to be 2.96E-24 Pa m^3^/mol using the HENRYWIN model within EPI Suite (v 4.11, Syracuse Research Corporation, USA).

Initially, the distribution of tigecycline to the lung compartments was assumed to occur primarily via passive diffusion, with the key parameter being the effective lung passive permeability. Due to the absence of direct permeability data for tigecycline, the reported Caco-2 cell permeability (20.76 × 10^−6^ cm/s) of tetracycline (34), a structural analog of tigecycline, was used as a surrogate. To enhance accuracy, this value was calibrated using propranolol permeability data. Specifically, the calibration factor (~1.66) was derived from the ratio of measured propranolol permeability from the same *in vitro* study (71.17 × 10⁻⁶ cm/s) to the reference value provided in Simcyp (43 × 10⁻⁶ cm/s). Applying this factor to the measured tetracycline permeability yielded a calibrated Caco-2 permeability of 12.54 × 10⁻⁶ cm/s for tigecycline. This value was subsequently used to estimate tigecycline’s permeability in Calu-3 cells (26), resulting in a predicted value of 7.83 × 10⁻⁶ cm/s. The Calu-3 permeability was further extrapolated to estimate tigecycline’s effective passive permeability in the lung, which was estimated to be 19.77 cm/s within Simcyp. Subsequently, active transport was also explored in lung compartments to match the observed lung (e.g., ELF) concentration in healthy volunteer studies. The unbound fraction of tigecycline in the pulmonary fluid (*f*_*u*, fluid_) was assumed to be one, given the low protein concentration in ELF (35). The unbound fraction in the pulmonary mass (*f*_*u*, mass_) for tigecycline was predicted in Simcyp based on the protein difference between plasma and lung tissue. Lung metabolism was assumed to be negligible.

The final parameters for the tigecycline lung PBPK model are displayed in Table 1. Based on this model, the simulated plasma and lung concentration–time profiles of tigecycline were compared with clinical observations from healthy adult studies to assess the model’s predictive performance.

### Effect of pulmonary pH alterations on tigecycline lung exposure

For healthy individuals, the pulmonary pH is mildly acidic, averaging around 6.6 (36). However, in pulmonary diseases, such as pneumonia, the pulmonary pH can be further reduced, with the most acidic value recorded at 5.6 in bacterially infected pneumonia patients (37). To represent these physiological alterations, a baseline pulmonary pH value of 6.6 was assumed for both ELF and ACs under healthy conditions. A range of pH values was tested to investigate the impact of pulmonary pH changes, with reductions from 6.6 to 5.6 in steps of 0.2 to simulate the effect of pneumonia-induced physiological alterations. Simulations were then conducted in default Sim-Healthy Volunteers (aged 25–65 years) using the Simcyp simulator, under the standard dosing regimen for tigecycline, to evaluate the impact of pH alteration on tigecycline lung exposure in both ELF and ACs. Additionally, observed lung concentrations from clinical PK studies in pneumonia patients were compared with the simulated results under corresponding clinical scenarios to verify the effect of pulmonary pH alterations on tigecycline lung exposure.

### Evaluation of tigecycline dosing regimens

Three clinically used tigecycline dosing regimens were evaluated: (i) the standard dose, with a loading dose of 100 mg followed by 50 mg every 12 hours; (ii) the high dose, with a loading dose of 200 mg followed by 100 mg every 12 hours; and (iii) the median dose, with a loading dose of 150 mg followed by 75 mg every 12 hours. For all three regimens, tigecycline was administered intravenously over 30 minutes for a duration of seven days. Three different pulmonary pH conditions were simulated: a healthy pulmonary pH of 6.6, the most acidic condition observed in pneumonia patients (pulmonary pH = 5.6), and an intermediate condition (pulmonary pH = 6.0).

In each simulation, a total of 100 virtual subjects (50% female), aged from 25 to 65 years, were generated using the Sim-Healthy Volunteers population within Simcyp. Ten trials, each with 10 subjects, were conducted to account for variability in patient characteristics. The steady-state AUC_0-24h_ of the simulated concentration–time curves in the lung was calculated for each individual, and the probability of target attainment (PTA) of tigecycline achieving its pharmacokinetic/pharmacodynamic (PK/PD) target of the ratio of the area under the curve up to 24  hours to minimal inhibitory concentration (AUC_0-24h_/MIC) ≥4.5 (38–40) was calculated across a MIC distribution range of 0.125 to 32 mg/L for both ELF and ACs (41, 42).

## RESULTS

### Clinical PK data for tigecycline

A total of 11 clinical PK studies on tigecycline were identified, including eight studies conducted in healthy volunteers and three studies involving pneumonia patients. The healthy volunteer studies included both single- and multiple-dose intravenous infusions, while the pneumonia patient studies focused on multiple-dose regimens (13, 40, 43–51). Tigecycline plasma concentration–time data were provided in all 11 studies, with five studies (three in healthy volunteers and two in pneumonia patients) also reporting PK data for ELF, and three studies (two in healthy volunteers and one in pneumonia patients) reporting data for ACs. Most of the data were presented as mean values with corresponding standard deviations. For the single-dose studies in healthy volunteers, tigecycline was administered in doses ranging from 12.5 to 300 mg, with infusion durations varying from 30 minutes to 240 minutes. For the multiple-dose studies in healthy volunteers, dosing regimens varied widely. Two primary tigecycline regimens were used in pneumonia patient studies, including the standard dose (100 mg loading dose followed by 50 mg every 12 hours) and the high-dose regimen (200 mg loading dose followed by 100 mg every 12 hours), with the latter being more commonly applied in elderly patients. A summary of the collected tigecycline PK studies is presented in Table 2.

**TABLE 2:** Summary of clinical pharmacokinetics studies on tigecycline^*a*^^,*b*^

Study	Demographics			Dose regimen	Site	Pharmacokinetics	
	*N*	Subject type	Age (years)			C_max_ (mg/L)	AUC (mg h/L)^*c*^
Muralidharan *et al.* (49)	6	Healthy	(18–44)	12.5/25/50/75/100/200/200 mg over 60 mins	Plasma	0.11/0.25/.38/0.57/.91/1.64/1.53	0.75/2.26/2.56/3.66/6.40/12.43/11.72^*d*^
				200/300 mg over 4 hrs	Plasma	0.68/0.96	14.24/16.73^*d*^
				25/50/100 mg over 60 mins q12h	Plasma	0.32/0.62/1.17	1.48/3.07/4.98
Zimmerman *et al.* (51)	20	Healthy	36.6 (27–45)	100 mg over 30 mins	Plasma	1.64 ± 0.28	2.48 ± 0.38^*e*^
Yamashita *et al.* (50)	8	Healthy	(20–45)	25 mg over 60 mins	Plasma	0.20 ± 0.05	0.82 ± 0.36
				50 mg over 60 mins	Plasma	0.40 ± 0.05	1.93 ± 0.44
				100 mg over 60 mins	Plasma	0.92 ± 0.13	5.02 ± 0.80
				150 mg over 60 mins	Plasma	1.52 ± 0.16	8.58 ± 1.76
Korth-Bradley *et al.* (46)	23	Healthy	46 (31–60)	100 mg over 60 mins	Plasma	0.98 ± 0.54	3.749 ± 1.32
Korth-Bradley *et al.* (48)	6	Healthy	53.83 (44–75)	100 mg over 60 mins	Plasma	0.60 ± 0.24	3.330 ± 0.71
Korth-Bradley *et al.* (47)	46	Healthy	37.3 (22–53)	50 mg over 30 mins	Plasma	0.43	2.37
				200 mg over 30 mins	Plasma	1.96	8.24
Conte *et al.* (43)	30	Healthy	33.6 (24.3–42.9)	100 mg followed by 50 mg q12h over 30 mins	Plasma	0.72 ± 0.24	1.73 ± 0.64
					ELF	0.37 ± 0.36	2.28
					AC	15.2 ± 7.6	134
Gotfried *et al.* (45)	17	Healthy	40 (30–50)	100 mg followed by 50 mg q12h over 30 mins	Plasma	0.98 ± 0.21	2.20 ± 0.42
					ELF	0.55 ± 0.50	NA
Burkhardt *et al.* (13)	9	Pneumonia patient	(25–62)	100 mg followed by 50 mg q12h	Plasma	0.54 ± 0.34	3.4 ± 2.1^*d*^
De Pascale *et al.* (40)	32	Pneumonia Patient	56 (46–68.5)	200 mg followed by 100 mg q12h over 30 mins	Plasma	0.34 [0.15–1.03]	3.61 [2.55–10.39]^*f*^
					ELF	0.42 [0.15–1.2]	NA
Dimopoulos *et al.* (44)	9	Pneumonia patient	69 (57–81)	200 mg over 1 hr followed by 100 mg q12h over 30 mins	Plasma	1.99 ± 1.82	12.89 ± 17.25

^*a*^ Data are presented as mean, mean (range), mean ± standard deviation, or median [interquartile range].

^*b*^ ACs, alveolar cells; AUC, area under the concentration-time curve; Cmax, maximum concentration; ELF, epithelial lining fluid; NA, not available.

^*c*^ AUC from 0 to infinity (AUC_0-inf_) for single-dose studies or from 0 to 12 hours at steady state (AUC_0-12h, ss_) for multiple-dose studies.

^*d*^ AUC here refers to AUC from 0 h to the last quantifiable concentration.

^*e*^ AUC here refers to AUC_0-12h_.

^*f*^ AUC here refers to AUC_0-24h_.

### Tigecycline PBPK model development and verification

#### Tigecycline basic PBPK model

**Fig 1:**
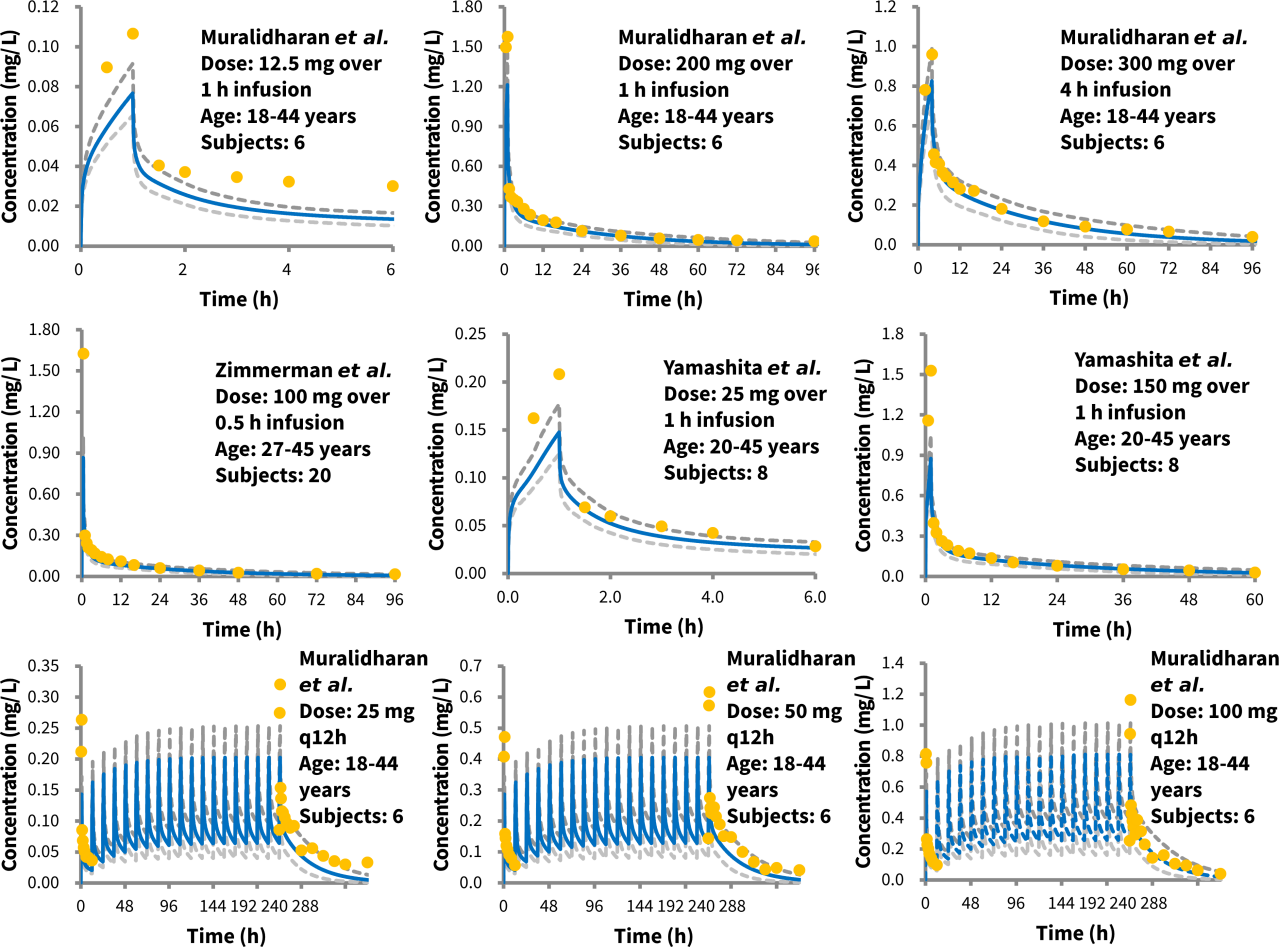
Observed versus predicted plasma concentration–time profiles in healthy adults following different intravenous dosing regimens of tigecycline. Orange dots represent mean observed data, and blue and grey lines represent the mean and 90% predicted interval of simulated plasma concentration–time profiles using the basic PBPK model. Data sources are presented in Table 2.

The basic PBPK model for tigecycline successfully captured the observed plasma concentration–time profiles in healthy volunteers following both single and multiple doses (Fig. 1; Fig. S2). The majority of the population concentration data points fell within the 90% prediction interval of the simulated concentration–time profiles, with approximately 6.82% (21 out of 308 data points) deviating from the two-fold range of the mean predicted values (Fig. S2). The simulated PK parameters *C*_max_ and AUC mostly fell within two-fold range of the observed values under single- and multiple-dose studies (Table S1), demonstrating the acceptable prediction performance of the basic model.

#### Tigecycline lung PBPK model

**Fig 2:**
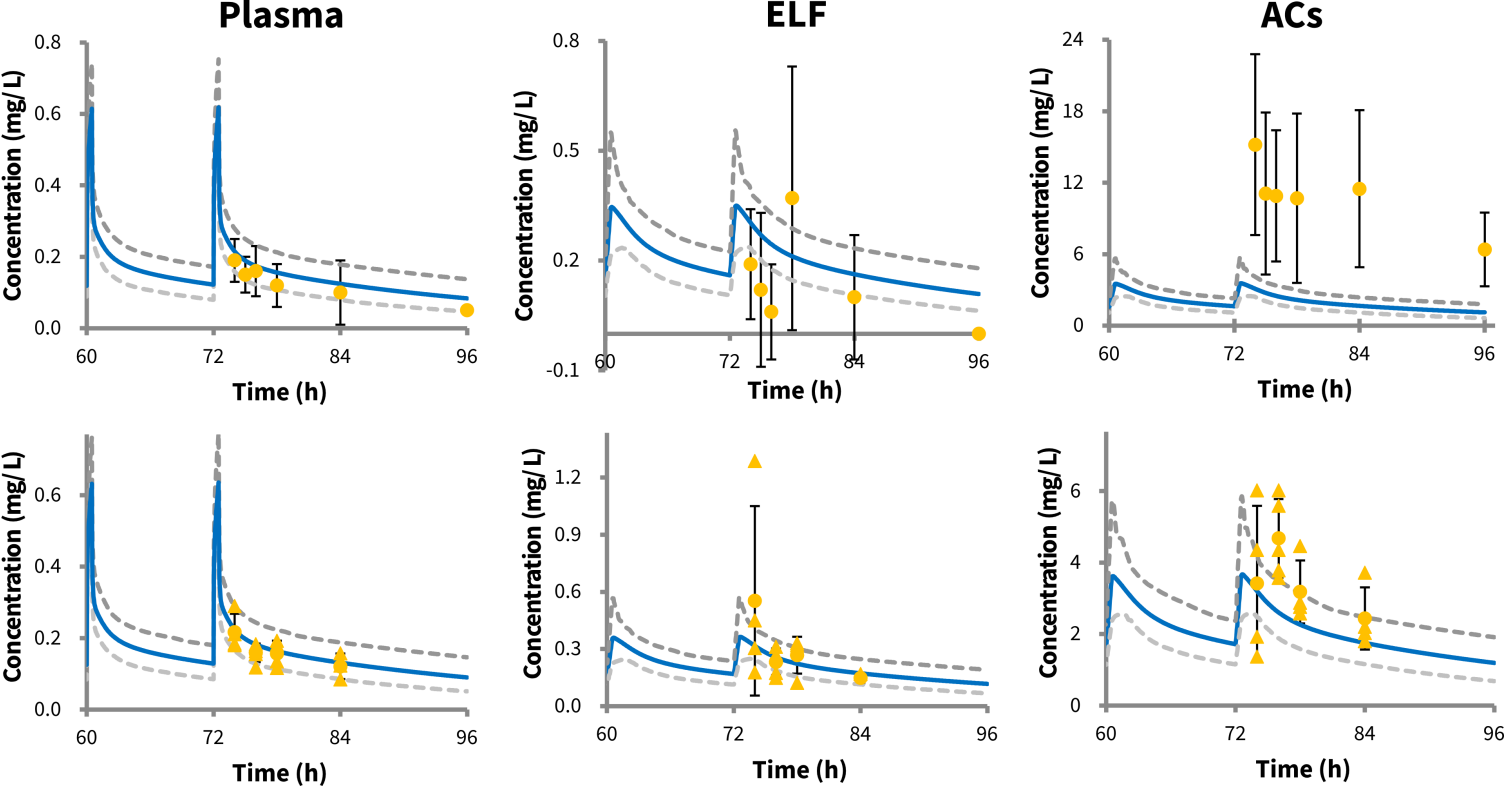
Observed versus predicted plasma, epithelial lining fluid (ELF), and alveolar cells (ACs) concentration–time profiles in healthy adults following the standard-dose tigecycline regimen. Orange dots represent mean observations with standard deviation indicated by black lines, and orange triangles represent individual values. Blue and grey lines represent the mean and 90% predicted interval of simulated concentration–time profiles using the multicompartment permeability-limited lung PBPK model. Data sources: Conte *et al.* (43) (upper panels) and Gotfried *et al.* (45) (bottom panels).

Based on physiological knowledge and clinical observations, an apical efflux transporter was incorporated into the lung PBPK model, with an optimized intrinsic clearance value of 2.5 µL/min/cm² (Table 1). The inclusion of this active transport process improved the accuracy of predicting tigecycline concentrations in the lung, particularly in ELF, compared to the model without the transporter (Fig. S3). Overall, the lung PBPK model was able to reasonably capture tigecycline exposure in both plasma and lung compartments in healthy adult studies, with most of the individual and population concentration data points falling within the 90% prediction interval of the simulated concentration–time profiles (Fig. 2). However, the model slightly underpredicted AC tigecycline concentrations in the study by Conte *et al.* (43).

### Effect of pulmonary pH alterations on tigecycline lung exposure

**Fig 3:**
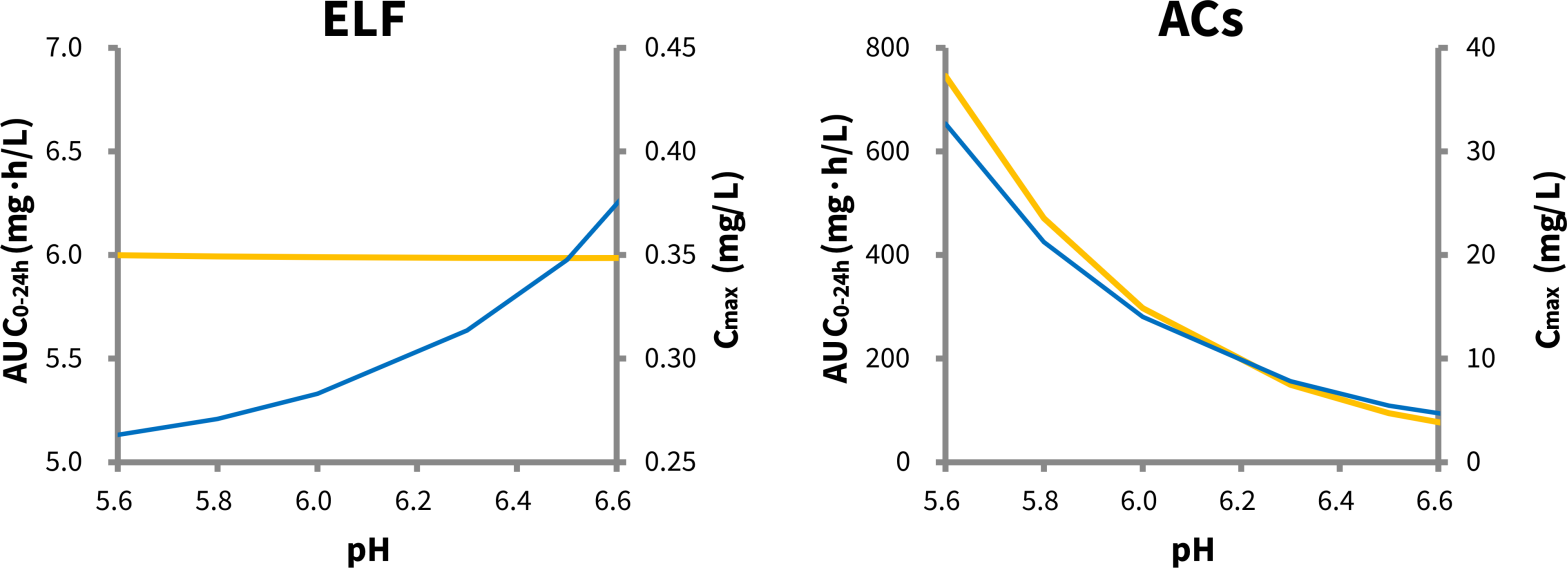
Changes in predicted tigecycline steady-state pharmacokinetics parameters (AUC_0-24h_ in yellow and *C*_max_ in blue) in epithelial lining fluid (ELF) and alveolar cells (ACs) following the standard-dose regimen at pulmonary pH values ranging from 5.6 to 6.6.

Pulmonary pH alteration showed a negligible impact on tigecycline exposure in the ELF, but it significantly influenced tigecycline concentrations in ACs (Fig. 3). Specifically, as the pulmonary pH decreased from 6.6 (healthy condition) to 5.6 (indicating infection), the steady-state AUC_0-24h_ for tigecycline under the standard dosing regimen increased slightly by 0.22% (from 5.99 mg∙h/L to 6.00 mg∙h/L) in ELF, while in ACs, it increased substantially by 12.39-fold (from 60.17 mg∙h/L to 745.79 mg∙h/L). Conversely, the *C*_max_ of tigecycline decreased by 34.50% (from 0.40 mg/L to 0.26 mg/L) in ELF, whereas in ACs, it increased markedly by 8.14-fold (from 4.01 mg/L to 32.69 mg/L) under the same pH shift from healthy to infected conditions. These results indicate that tigecycline exposure in ACs is more sensitive to alterations in pulmonary pH during bacterial infection compared to ELF.

**Fig 4:**
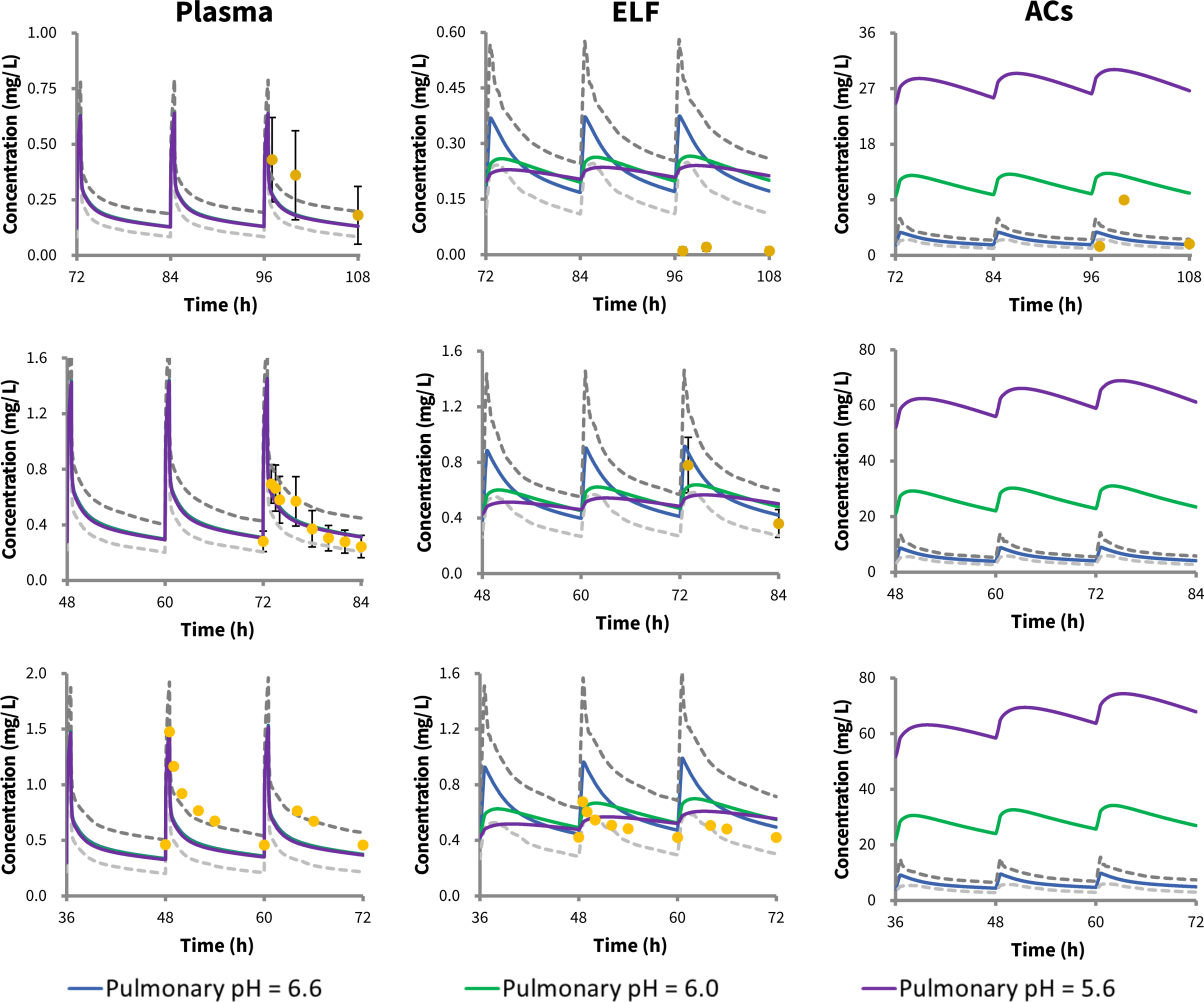
Observed versus predicted plasma, epithelial lining fluid (ELF), and alveolar cells (ACs) concentration–time profiles in pneumonia patients following different tigecycline regimens. Orange dots represent mean observed data with standard deviation indicated by black lines. The blue, green, and purple lines represent mean simulated concentration–time profiles at the pulmonary pH of 6.6, 6.0, and 5.6, respectively. The grey lines represent the 90% predicted interval of simulated concentration–time profiles at pH 6.6. Data sources: Burkhardt *et al.* (13) (upper panels), De Pascale *et al.* (40) (middle panels), and Dimopoulos *et al.* (44) (bottom panels).

Three published tigecycline PK studies provided lung concentration data in pneumonia patients, with three reporting data for ELF and one for ACs (13, 40, 44). For all three studies, our lung PBPK model could adequately describe the tigecycline concentrations in plasma (Fig. 4). In terms of tigecycline concentrations in ELF, the observations fall within the 90% predicted interval of the simulations, regardless of the pH alterations, except for the study by Burkhardt *et al.* (13), where our lung PBPK model showed an overprediction, even under the most acidic condition (pulmonary pH = 5.6). Notably, for the tigecycline concentrations in ACs, our prediction aligned well with observed values at a pulmonary pH of 6.6, as two of the three data points fell within the 90% prediction interval. Due to the lack of observed concentration data in ACs, model predictions in ACs under three different pH conditions (pH = 6.6, 6.0, and 56) are presented for the patient studies, as depicted in Fig. 4.

### Evaluation of tigecycline dosing regimens

For all the evaluated doses, tigecycline AUC exposure in ELF remained relatively stable, showing only negligible decreases, while in ACs, tigecycline AUC exposure increased more than 12.39-fold for standard, median, and high doses, when transitioning from healthy conditions (pulmonary pH = 6.6) to pneumonia conditions (pulmonary pH = 5.6) (Table 3; Fig. S4).

**TABLE 3:** Predicted mean tigecycline pharmacokinetic parameters in the lung under different pH conditions (healthy: 6.6; in-between: 6.0; infection: 5.6) for three clinical dosing regimens^*a*^

Dose regimen	Pulmonary pH = 6.6		Pulmonary pH = 6.0		Pulmonary pH = 5.6	
	AUC_0-12h, ss_ (mg h/L) in ELF	AUC_0-12h, ss_ (mg h/L) in ACs	AUC_0-12h, ss_ (mg h/L) in ELF	AUC_0-12h, ss_ (mg h/L) in ACs	AUC_0-12h, ss_ (mg h/L) in ELF	AUC_0-12h, ss_ (mg h/L) in ACs
Standard dose	2.99	30.09	3.00	148.86	3.01	372.90
Median dose	4.49	45.13	4.50	223.29	4.52	559.34
High dose	5.99	60.17	6.00	297.72	6.02	745.79

^*a*^ AUC_0-12h, ss_, area under the concentration–time curve from 0 to 12 hours at steady state; ELF, epithelial lining fluid; ACs, alveolar cells; standard dose, a loading dose of 100 mg tigecycline followed by 50 mg every 12 hours; median dose, a loading dose of 150 mg tigecycline followed by 75 mg every 12 hours; high dose, a loading dose of 200 mg tigecycline followed by 100 mg every 12 hours.

**Fig 5:**
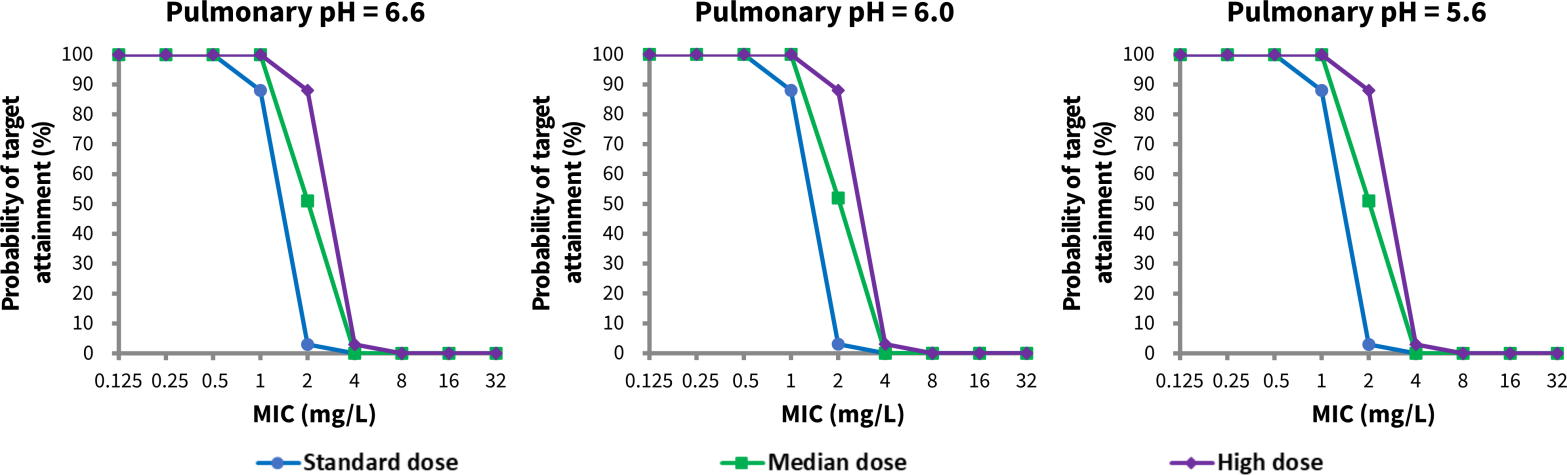
Probability of tigecycline PKPD target (AUC_0-24h_/MIC ≥4.5) attainment in epithelial lining fluid (ELF) following the standard (blue), median (green), and high (purple) doses of tigecycline at pulmonary pH values of 6.6 (left), 6.0 (middle), and 5.6 (right panel), respectively. The standard, median, and high doses of tigecycline represent a loading dose of 100, 150, and 200 mg, followed by 50, 75, and 100 mg every 12 hours, respectively.

Based on these simulated tigecycline lung exposures, particularly in ELF, the PTA was calculated under a range of MIC values (0.125 to 32 mg/L), which encompasses the MIC distributions of several common pathogens responsible for CAP and HAP. For pathogens with an MIC ≤1 mg/L, all three clinical dosing regimens (standard, median, and high dose) achieved a PTA ≥90%, suggesting a potentially favorable clinical response. However, when treating resistant pathogens with an MIC of 2 mg/L, the standard and median dosing regimens of tigecycline were unable to reach a preferable PTA, particularly the standard dose, which resulted in a PTA in ELF of around 3% regardless of the pulmonary pH values. In contrast, the high-dose regimen was still able to achieve a satisfactory PTA, with a stable PTA of around 88% at pulmonary pH values ranged from 6.6 to 5.6. As pathogen resistance increased further, with MIC values >2 mg/L, all three dosing regimens failed to achieve an adequate PTA, with all the predicted PTA falling below 5%, regardless of the pulmonary pH condition (Fig. 5).

## DISCUSSION

In this study, we developed a lung PBPK model for tigecycline, which reasonably described tigecycline exposure in pneumonia patients, particularly in ELF and ACs. Our model demonstrated that alterations in pulmonary pH, which commonly occur during bacterial pneumonia, did not significantly affect tigecycline AUC exposure in ELF but resulted in substantial accumulation in ACs. Furthermore, our simulations indicated that the standard dose of tigecycline is adequate for treating infections caused by susceptible isolates, but higher doses may be necessary for resistant bacteria, especially extracellular pathogens.

During the development of the tigecycline lung PBPK model, our initial model did not account for active transport between the lung mass and ELF, which led to an underprediction of tigecycline concentrations in ELF but reasonable capture of concentrations in ACs, when compared to the observed data from the healthy adult study by Gotfried *et al.* (45). To improve the simulated concentrations in ELF, an apical efflux transporter was incorporated to account for active transport processes, particularly in the lung apical membranes where the P-glycoprotein (P-gp) is typically expressed (52–54), as previous *in vitro* studies have demonstrated that tigecycline is a substrate of p-gp (55). The inclusion of this apical efflux transporter significantly improved the model’s ability to predict tigecycline concentrations in both the ELF and ACs, aligning more closely with the observations from Gottfried *et al.* (Fig. S3) (45). Moreover, this adjustment led to a reasonable prediction of ELF concentrations in the study by Conte *et al. (43*). These findings confirm that the incorporation of the P-gp transporter enhanced the accuracy of our model’s predictions, bringing them into closer alignment with *in vivo* data. Similarly, a previous PBPK model for pyrazinamide, which also included an efflux transporter between the lung mass and ELF, successfully addressed the underprediction in ELF concentrations (26).

Despite the improvement in model predictions with the inclusion of active apical efflux from lung mass to ELF, discrepancies were observed between our simulation results and those from the healthy adult study by Conte *et al.*, where we found an underprediction of tigecycline concentrations in ACs (43). This discrepancy may be due to variations in pulmonary pH across healthy populations. In our study, we adapted a default pulmonary pH of 6.6 for healthy adults, which is based on the average published measurements (5.7–7.5) (37), but this value can vary significantly between individuals. Sensitivity analysis revealed that adjusting the pulmonary pH in the healthy population to 6.0 improved the model’s alignment with observed concentrations in both ELF and ACs (Fig. S5), supporting the notion that pulmonary pH variability contributes to discrepancies across studies. Furthermore, differences in methodologies (such as the BAL sampling lobes) used to measure drug concentrations in the lung, as well as potential variations in different populations (such as smoking status and clinical conditions) (45), may explain some of the observed discrepancies.

Previous studies have demonstrated that albumin concentrations in ELF are substantially lower than in serum, averaging 8.8% of plasma albumin levels (56). Although certain lung diseases may elevate ELF protein levels, the increase is typically less than twofold (35, 57). Given the very low albumin concentrations in the ELF, we assumed that tigecycline’s protein binding in ELF would be negligible (i.e., *f*_*u*,fluid_ = 1), despite its moderate plasma protein binding reported in previous studies (28). To assess the impact of this assumption, we conducted a sensitivity analysis by varying *f*_*u*,fluid_ from 0.4 to 1.0 using the standard tigecycline dose in the Sim-Healthy Volunteers population. The results showed no effect on free tigecycline concentrations in either ELF or ACs (Fig. S6). Since only the unbound fraction is pharmacodynamically active and the ELF protein content is minimal, the assumption of *f*_*u*,fluid_ = 1 in the final PBPK model is considered reasonable. Nevertheless, this assumption should be interpreted with caution due to the absence of experimental data on tigecycline protein binding in pulmonary fluids.

Our study revealed that pH alterations in the lung have a more pronounced effect on tigecycline exposure in ACs than in the ELF, likely due to increased lysosomal trapping under acidic conditions. Given tigecycline’s physicochemical properties, including moderate basicity and lipophilicity, it is susceptible to ion trapping in acidic lysosomes (23). This suggests that for pathogens residing in intracellular spaces, such as those in ACs, the pneumonia-induced acidic microenvironment may lead to accumulated drug exposure and potential toxicity. In contrast, changes in pulmonary pH have minimal impact on the drug’s effectiveness for extracellular pathogens found in ELF.

In clinical pneumonia scenarios, our lung PBPK model adequately described tigecycline concentrations in ELF in two of the three published PK studies, and it also captured the limited data available for ACs in one study. Importantly, the optimal pH values varied across studies where the best model performance was achieved, highlighting the variability and heterogeneity among patients. This further reinforces the importance of considering patient-specific factors when determining optimal treatment regimens. Specifically, the study by Burkhardt *et al*. reported very low tigecycline concentrations in ELF, which our model did not capture accurately (13). These extremely low concentrations (0.01–0.02 mg/L) contrast sharply with the higher concentrations observed in the other two studies (0.36–0.78 mg/L). The discrepancies may be attributable to differences in methodological approaches and physiological changes specific to HAP patients. Further studies are needed to resolve these discrepancies and understand the reasons for potential differences in intrapulmonary concentrations, particularly in HAP populations.

Our PK/PD analysis, based on AUC_0-24h_/MIC ≥4.5 in ELF (38–40), revealed that the standard dose of tigecycline is sufficient to achieve a favorable clinical response for susceptible pathogens (MIC ≤1 mg/L), but may fail for resistant pathogens with MIC values ≥2 mg/L. In particular, our simulations showed that a high-dose regimen (200 mg loading dose followed by 100 mg every 12 hours) is required to achieve adequate target attainment for pathogens with a MIC of 2 mg/L, while the standard and median doses fail to meet this target. These findings are consistent with previous studies suggesting that high-dose tigecycline can improve clinical outcomes and reduce mortality in patients with HAP, particularly when caused by resistant organisms (12, 14, 15). Notably, for pneumonia (3, 58), common pathogens, such as *Acinetobacter baumannii*, *Klebsiella pneumoniae*, *Streptococcus aureus*, and *Haemophilus influenzae*, are primarily extracellular pathogens located in the ELF, in which the MIC_90_ (the lowest concentration of an antimicrobial agent that inhibits the visible growth of 90% of a standardized microbial population under specific *in vitro* conditions) value for two major causes (*Acinetobacter baumannii* and *Klebsiella pneumoniae*) of HAP is 2 mg/L according to EUCAST (11, 59, 60). This highlighted the necessity of applying high-dose tigecycline in treating patients with HAP.

There are several limitations to this study. Firstly, the availability of clinical BAL data is limited, resulting in a lack of comprehensive lung exposure data for tigecycline in both healthy adults and pneumonia patients. Additionally, differences in BAL techniques across study sites, along with inherent variability within and between patient populations (particularly among elderly pneumonia patients), contribute to substantial differences in available lung exposure data. Despite these limitations, *in silico* approaches, such as the lung PBPK model, offer valuable insights into drug exposure and can help evaluate potential efficacy based on site-specific concentrations. Furthermore, in this study, we focused exclusively on the effect of pulmonary pH changes in bacterial infections. Other pathophysiological alterations, such as changes in pulmonary permeability caused by the local inflammatory response (21), may also influence tigecycline behavior in the lung but were not explored here due to the limited availability of quantitative data on altered pulmonary permeability. Future studies are warranted to validate our findings in larger and more diverse patient populations, as well as to refine the model with additional physiological factors and clinical data.

In conclusion, our lung PBPK model provides a valuable tool for understanding tigecycline pharmacokinetics in pneumonia patients, particularly with respect to drug exposure in the ELF and ACs. Our findings showed that alterations in pulmonary pH have a more pronounced impact on tigecycline AUC exposure in ACs compared to ELF. Our analyses underscored the importance of optimizing tigecycline dosing for lung infections caused by resistant pathogens. The use of PBPK modeling in clinical decision-making can improve our understanding of drug behavior in the lungs, ultimately supporting more effective treatment strategies for pneumonia and other respiratory infections.

## SUPPLEMENTAL MATERIAL

Supplemental material aac.00004-25-s0001.pdf Table S1; Fig. S1 to S6.

ASM does not own the copyrights to Supplemental Material that may be linked to, or accessed through, an article. The authors have granted ASM a non-exclusive, world-wide license to publish the Supplemental Material files. Please contact the corresponding author directly for reuse.
