# Supplementary material for: Evaluating tigecycline dosing for hospital-acquired pneumonia patients: insights from physiologically-based pharmacokinetic modeling of lung exposure
Source: Antimicrob Agents Chemother. 2025 May 20;69(7):e00004-25. doi: 10.1128/aac.00004-25 (PMC12217487; doi:10.1128/aac.00004-25)
Supplement: Supplemental material — Table S1; Fig. S1 to S6. [file aac.00004-25-s0001.pdf]

Table S1. Comparison of observed and predicted mean pharmacokinetic parameters for tigecycline in single and multiple dose studies in healthy adults. Data are presented as mean or mean (range).

Study	Age (years)	Dose regimen	Site	C_{max} (mg/L)			AUC (mg·h/L)		
				Prediction	Observation	Ratio (Pred./Obs.)	Prediction	Observation	Ratio (Pred./Obs.)
Muralidharan *et al.* (2005)	(18-44)	12.5/ 25/ 50/ 75/ 100/ 200/ 200 mg over 60 mins	Plasma	0.08/ 0.15/ 0.31/ 0.46/ 0.61/ 1.22/ 1.17	0.11/ 0.25/ 0.38/ 0.57/ 0.91/ 1.64/ 1.53	0.69/ 0.61/ 0.81/ 0.81/ 0.67/ 0.74/ 0.77	0.56/ 1.12/ 2.23/ 3.35/ 4.46/ 8.92/ 8.78*	0.75/ 2.26/ 2.56/ 3.66/ 6.40/ 12.43/ 11.72*	0.74/ 0.49/ 0.87/ 0.91/ 0.70/ 0.72/ 0.75
		200/ 300 mg over 4 h		0.55/ 0.83	0.68/ 0.96	0.81/ 0.86	8.92/ 13.26*	14.24/ 16.73*	0.63/ 0.79
		25/ 50/ 100 mg q12h over 60 mins		0.20/ 0.40/ 0.81	0.32/ 0.62/ 1.17	0.63/ 0.65/ 0.69	1.09/ 2.18/ 4.35	1.48/ 3.07/ 4.98	0.74/ 0.71/ 0.87
Zimmerman *et al.* (2007)	36.6 (27-45)	100 mg over 30 mins	Plasma	0.87	1.64	0.53	1.89**	2.48**	0.76
Yamashita *et al.* (2014)	(20-45)	25/ 50/ 100/ 150 mg over 30 mins	Plasma	0.15/ 0.29/ 0.59/ 0.88	0.20/ 0.40/ 0.92/ 1.52	0.73/ 0.74/ 0.64/ 0.58	0.89/ 1.06/ 3.18/ 5.71	0.82/ 1.93/ 5.02/ 8.58	1.08/ 0.55/ 0.63/ 0.67
Korth-Bradley *et al.* (2011)	46 (31-60)	100 mg over 60 mins	Plasma	0.64	0.98	0.65	4.33	3.75	1.16
Korth-Bradley *et al.* (2012)	53.83 (44–75)	100 mg over 60 mins	Plasma	0.64	0.60	1.06	4.52	3.33	1.36
Korth-Bradley *et al.* (2013)	37.3 (22-53)	50/ 200 mg over 30 mins	Plasma	0.43/ 1.73	0.43/ 1.96	1.01/ 0.88	2.15/ 8.60	2.37/ 8.24	0.91/ 1.04
Conte *et al.* (2005)	33.6 (24.3-42.9)	100 mg then 50 mg q12h over 30 mins	Plasma/ ELF/ ACs	0.62/ 0.35/ 3.56	0.72/ 0.37/ 15.20	0.86/ 0.95/ 0.23	2.19/2.72/ 27.74	1.73/ 2.28/ 134.00	1.27/ 1.19/ 0.21
Gotfried *et al.* (2017)	38 (27-47)	100 mg then 50 mg q12h over 30 mins	Plasma	0.64	0.98	0.65	2.29	2.20	1.04

AUC: area under the concentration-time curve from 0 to infinity ($AUC_{0\text{-}inf}$) for single dose studies or from 0 to 12 hours at steady state ($AUC_{0\text{-}12,\,ss}$) for multiple dose studies, ELF: epithelial lining fluid, ACs: alveolar cells. *AUC here refers to AUC from 0 h to the last quantifiable concentration; **AUC here refers to $AUC_{0\text{-}12h}$.

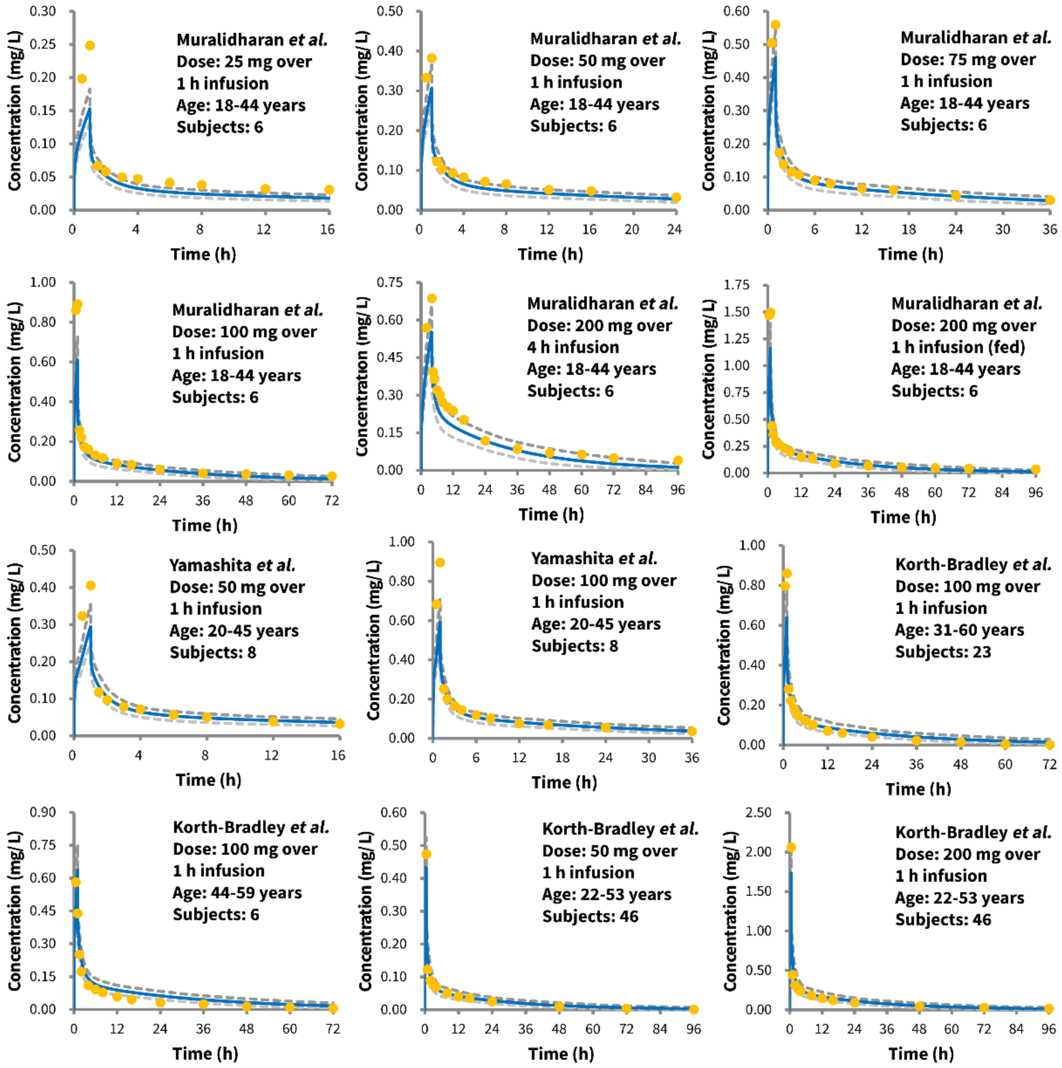

Figure S1. Observed versus predicted tigecycline plasma concentration-time profiles in healthy adults following different intravenous dosing regimens of tigecycline (extended data from Figure 1). Orange dots represent mean observed data, blue and grey lines represent the mean and 90% predicted interval of simulated plasma concentration-time profiles using the basic PBPK model. Data sources are presented in Table 2.

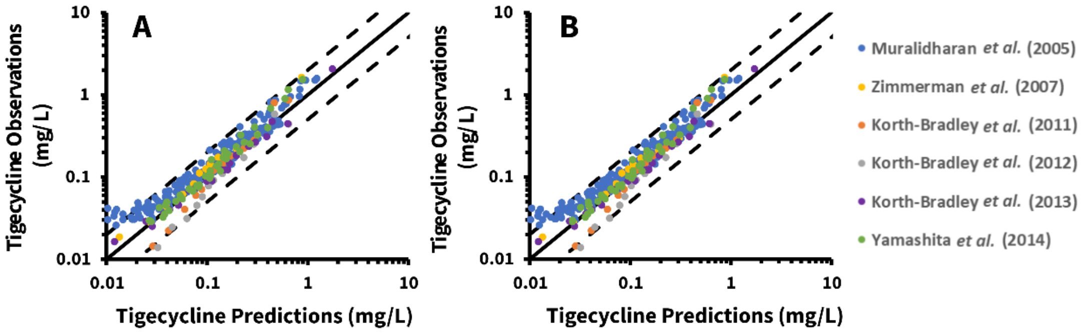

Figure S2. Observed versus predicted mean tigecycline plasma concentrations in healthy adult studies using the basic PBPK model (A) and multicompartment permeability-limited lung PBPK model (B). The solid black line represents the line of identity and the dashed black lines indicate the two-fold range. Literature data sources are presented in Table 2.

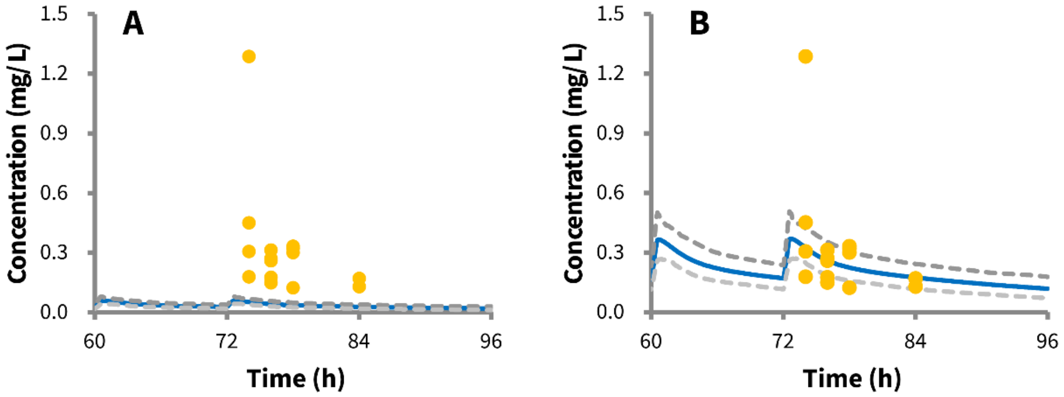

Figure S3. Observed versus predicted tigecycline epithelial lining fluid (ELF) concentration-time profiles in healthy adults following the standard-dose regimen. Orange dots represent individual observed data, blue and grey lines represent the mean values and 90% predicted interval of the simulated concentration-time profiles using the multicompartment permeability-limited lung PBPK model without (A) and with (B) an active efflux transporter. Data source: Gotfried *et al.* (2017).

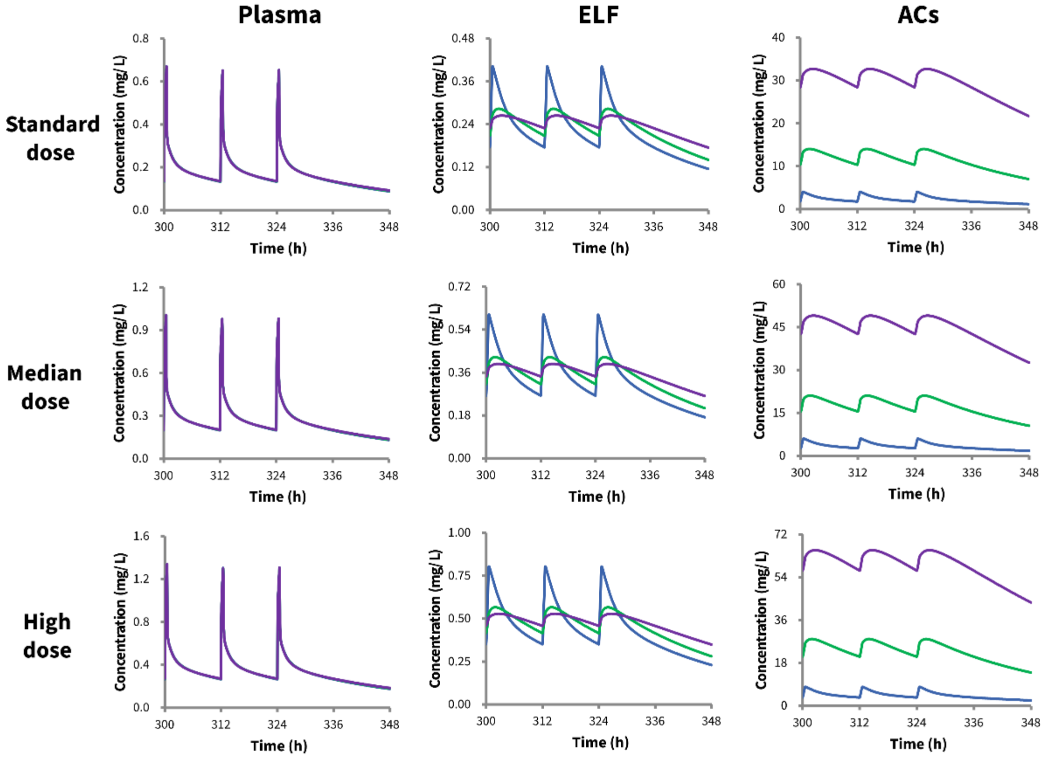

Figure S4. Simulated mean tigecycline concentration-time profiles in plasma, epithelial lining fluid (ELF) and alveolar cells (ACs) at the pulmonary pH values of 6.6 (blue), 6.0 (green) and 5.6 (purple) under standard (upper panels), median (middle panels), and high (bottom panels) dosing regimens. The standard, median, and high dose of tigecycline correspond to loading doses of 100, 150 and 200 mg, followed by 50, 75 and 100 mg every 12 hours, respectively.

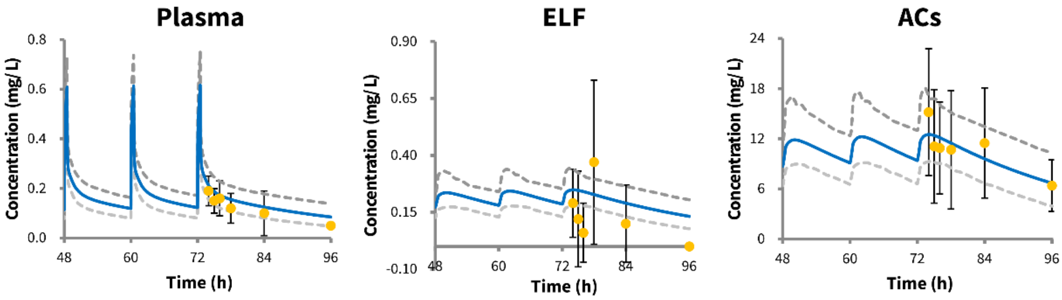

Figure S5. Observed versus predicted tigecycline plasma, epithelial lining fluid (ELF) and alveolar cells (ACs) concentration-time profiles in healthy adults at a pulmonary pH value of 6.0 under the standard-dose regimen. Orange dots represent mean observed data with standard deviation indicated by black lines. Blue and grey lines represent the mean values and 90% predicted interval of simulated concentration-time profiles using the multicompartment permeability-limited lung PBPK model. Data source: Conte *et al*. (2005).

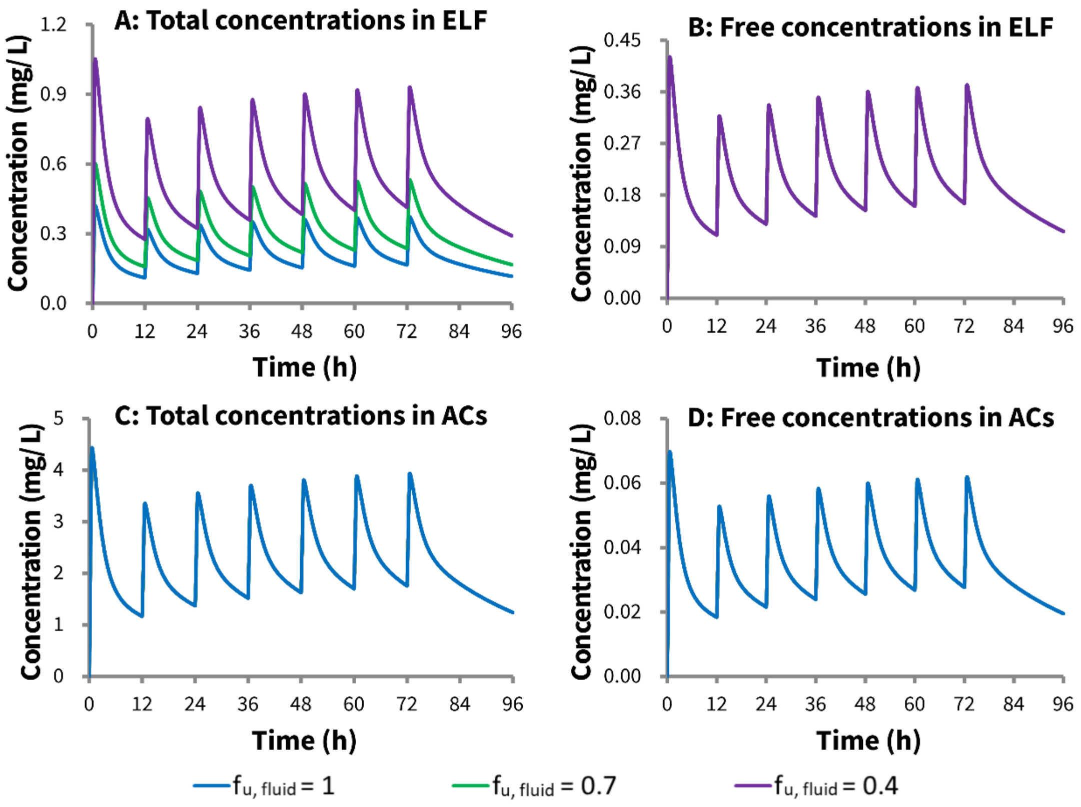

Figure S6. The total (A and C) and free (B and D) simulated concentration–time profiles of tigecycline in epithelial lining fluid (ELF) (upper panels) and alveolar cells (ACs) (bottom panels) under varying $f_{u,fluid}$ settings. The concentrations in panel B, C, and D under different $f_{u,fluid}$ values are overlapped each other.
